# Creosote in Dysentery

**Published:** 1846-01

**Authors:** 


*Creosote in Dysentery.*—In a severe form of dysentery which occurred near Tunbridge Wells, and in which all methods of treatment appeared unsuccessful, the mortality being as high as 25 per cent., Dr. Wilmot thought of trying creosote enemata in the strength of ʒi. to ℥xij. of starch. This remedy produced a speedy amelioration of the disease. *Ibid.*

